# Venous vessel-wall assessment with superb microvascular imaging in Behçet syndrome

**DOI:** 10.1136/rmdopen-2024-005382

**Published:** 2025-04-05

**Authors:** Yasemin Kayadibi, Yesim Ozguler, Melike Melikoglu, Sinem Nihal Esatoglu, Ugur Kimyon, Ayse Kalyoncu Ucar, Ibrahim Adaletli, Gulen Hatemi

**Affiliations:** 1 Cerrahpasa Faculty of Medicine, Department of Internal Medicine, Division of Radiology, Istanbul University-Cerrahpasa, Istanbul, Turkey; 2 Cerrahpasa Faculty of Medicine, Department of Internal Medicine, Division of Rheumatology, Istanbul University-Cerrahpasa, Istanbul, Turkey; 3 Behçet’s Disease Research Center, Istanbul University-Cerrahpasa, Istanbul, Turkey; 4 Cerrahpasa Faculty of Medicine, Department of Internal Medicine, Istanbul University-Cerrahpasa, Istanbul, Turkey

**Keywords:** Behcet Syndrome, Cardiovascular Diseases, Systemic vasculitis, Vasculitis

## Abstract

**Objectives:**

Superb microvascular imaging (SMI) provides a more sensitive assessment of small vessels compared with power and colour Doppler ultrasonography (PDUS and CDUS). We aimed to investigate the potential of SMI for use in research and clinical practice in Behçet syndrome (BS) and to gain a better understanding of erythema nodosum (EN) like and superficial thrombophlebitis (STM) lesions using SMI.

**Methods:**

We studied 51 patients with BS and 31 patients with non-BS with red palpable lesions on physical examination. B-mode US, CDUS, PDUS and SMI were performed and recorded by the same radiologist and recorded images were additionally evaluated independently by another radiologist. Vessel-wall signal intensity of STM lesions by SMI was graded in four groups according to the percentages of the affected vessel-wall area (Grade 0: no signal, Grade 1: < 25%, Grade 2: 25%–50%, Grade 3: 50%–75% and Grade 4: >75%).

**Results:**

The lesions of 26 patients with BS and 17 patients with non-BS were diagnosed as STM with CDUS. SMI of STM lesions revealed vessel-wall signals in 21/26 patients with BS (81%), in contrast to only 3/17 (18%) patients with non-BS. While 16 of 21 patients with BS (76%) had at least grade 2 signal, only 1 of 3 non-BS had it. Lesions of 25 BS and 11 non-BS were diagnosed as EN by CDUS and SMI showed no vessel-wall signal in these patients.

**Conclusions:**

Evaluation of the vessel wall with SMI in BS may be a promising approach for showing inflammation as an inexpensive, non-invasive and reliable imaging method that does not require a contrast agent.

**WHAT IS ALREADY KNOWN ON THIS TOPIC**

**WHAT THIS STUDY ADDS**

- This is the first imaging study to explore acutely inflamed vascular lesions in BS, demonstrating that vessel-wall vascularisation—an indicator of vessel-wall inflammation—is more frequent and more pronounced in acute superficial thrombophlebitis lesions caused by BS compared with those of non-BS aetiologies.
- SMI has potential for use in vessel-wall assessment in BS, visualising vascularisation that cannot be observed with conventional DUS.

**HOW THIS STUDY MIGHT AFFECT RESEARCH, PRACTICE OR POLICY**

- Further research is necessary to validate the use of SMI in patients with BS and various vascular lesions to examine the relationship among SMI findings, histopathology and disease activity, and to determine its role in guiding treatment decisions and in the differential diagnosis of patients with thrombosis.

## Introduction

 Vascular involvement is a major cause of morbidity and mortality in Behçet syndrome (BS).^1^ The prevalence of vascular involvement in BS ranges from 15% to 50%, with approximately 85% of these cases manifesting as venous thrombosis.^2^ Lower extremity deep vein thrombosis accounts for 70% of all vascular events in BS.^3^ The true prevalence of superficial thrombophlebitis (STM) is not clear because distinguishing STM from erythema nodosum (EN) by physical examination can be challenging. While STM is characterised by cord-like lesions accompanied by redness on the skin when a longer vein segment is involved, it may present as a palpable, hard and painful nodule under the skin resembling EN when a shorter vein segment is involved. Therefore, colour Doppler ultrasonography (CDUS) is crucial for differentiating these lesions.^4^ The most accurate way to determine the true prevalence of STM among patients with BS would be to examine all palpable, red lesions with CDUS in a large cohort, but we are not aware of any such study. Nonetheless, some studies have reported STM as the most common type of vascular involvement in BS, with frequencies reaching up to 55% of all patients with BS in certain retrospective cohorts.^5 6^

The exact mechanism of thrombosis in BS has not yet been fully elucidated. It is thought to be more of an inflammation-induced thrombosis rather than one caused by classic procoagulant factors. Endothelial dysfunction, immune cells (mainly neutrophils), oxidative stress and activated platelets are suggested to be responsible for inflammation-induced thrombosis in inflammatory conditions, such as autoimmune diseases, infection and malignancies. There are also a number of studies in BS showing that increased neutrophil activation leads to enhanced production of reactive oxygen species and neutrophil extracellular traps, and activates endothelial cells and platelets resulting in thrombosis.^7^,^11^ In addition to the above-mentioned mechanisms triggered by systemic inflammation, the vein wall itself is thought to be inflamed, promoting thrombosis even more in BS. This may explain the increased frequency of thrombosis compared with other inflammatory diseases and the sticky nature of the thrombus with poor recanalisation in BS.

Thrombosis is not always detected in STM lesions; superficial vein vessel-wall inflammation without thrombosis is referred to as phlebitis. These two types of lesions can be distinguished by CDUS. While STM is characterised by a non-compressible vein, wall thickening and intraluminal echogenicity, phlebitis is characterised by a fully or partially compressible vein with wall thickening, vessel-wall hypoechogenicity and lack of intraluminal echogenicity. These lesions were also defined histopathologically. Misago *et al* studied nodular lesions in patients with BS and found that some lesions had phlebitis without thrombosis but with neutrophils in the vessel lumen, while others showed phlebitis accompanied by thrombosis, indicating thrombophlebitis.^12^ This finding suggests that vessel-wall inflammation may be the initial pathology and the trigger for thrombosis in BS. While inflammatory cell infiltration, particularly around the vasa vasorum, has been observed in non-pulmonary arterial lesions in BS,^13^,^15^ no study has demonstrated this in venous lesions, likely due to the smaller size of venous vessels.

STM is a good elementary lesion to study the pathogenesis of vasculitis in BS since it is relatively more accessible for both imaging and histopathological examination. One major drawback, however, is its clinical similarity to EN. Several studies have examined the histopathology of nodular lesions of BS, without correlated imaging, to identify whether these are EN or STM.^1216^,^19^

Superb microvascular imaging (SMI) is a newly developed imaging modality to evaluate low-velocity blood flow that cannot be detected with classical conventional Doppler ultrasonography (US). It can visualise the blood flow in vessels as small as venules (<100 µm in diameter), showing vascularisation patterns, density and infinitesimal blood supply alterations in the tissue, thanks to its high frame rate (>50 frames/s) and high spatial resolution.^20^,^22^ With these advantages, SMI has proven useful in evaluating atherosclerotic plaque stability, thyroid nodules, breast masses, synovitis, intraductal lesions and liver tumours.^20 21 23 24^ A cross-sectional study, including 96 Takayasu arteritis patients, showed that SMI was superior to CDUS in differentiating active disease from inactive disease and in detecting neovascularisation in carotid arteries.^25^

Assuming that venous thrombosis in BS stems from venous wall inflammation, we hypothesised that SMI would show increased microcirculatory activity in the walls of thrombosed veins in BS in comparison with thrombosis caused by other conditions. For this purpose, we targeted STM lesions due to their frequent occurrence and relative ease of accessibility.

## Methods

This study was performed between March 2021 and February 2023. The institutional review board of our institution approved the study (179866–2021). Written informed consent was obtained from all patients.

### Patients

All patients with BS presenting with acute red palpable lesions to our BS outpatient clinic were invited to the study. Patients with non-BS were recruited from our rheumatology and internal medicine clinics. Grey-scale US***,*** CDUS, power Doppler US (PDUS) and SMI were performed on the same day in all of the patients. The radiologist (YK) recorded the localisation of the lesions. After imaging, we included patients with acute STM, acute phlebitis or EN in the study. We excluded patients with extensive varicose veins due to chronic DVT or those with chronic STM. We noted demographic features, BS manifestations, diagnosis and underlying causes in patients with non-BS and drugs of each patient. C reactive protein (CRP) levels of patients with BS were retrieved from hospital records at the nearest time point to the radiological examination.

### Imaging protocol

Grey-scale imaging was the first step in differentiating acute STM or phlebitis lesions from EN, chronic STM, DVT and non-specific lesions. The patients were evaluated by the same radiologist, who was unaware of the clinical diagnoses. First, the lesion area was evaluated by grey-scale US (Toshiba Aplio A US scanner) (Toshiba Medical Systems, Tokyo, Japan) with a superficial probe in the 12–16 MHz range. If a vein was detected, it was further investigated to determine whether it could be compressed by the probe in the axial plane and to assess the presence of wall thickening. A non-compressible vein with wall thickening and intraluminal echogenicity, suggesting thrombosis, was considered to be an acute STM. Total or partial compressibility of the vein accompanied by hypoechogenicity of the vessel wall without intraluminal echogenicity was considered to be acute phlebitis. Lesions that did not include a vascular structure were considered to be EN if there was increased echogenicity in the adipose tissue, suggesting panniculitis. Lesions were classified as chronic STM if the vein wall was thick, showed a partial response to compression and had linear intraluminal echogenicities adherent to the wall. Compressible, tortuous vessels with increased lumen width and wall thickness were considered as varicose veins.

The second step was to perform CDUS to investigate intraluminal colour coding and PDUS to assess colour coding in the vessel wall and adipose tissue. If the vein was not compressible on grey-scale ultrasound and no flow was detected by CDUS, the lesion was confirmed to be an acute STM. If the vein showed any degree of compression along with wall thickening, hypoechogenicity in the vessel wall on grey-scale US and the presence of vascularisation on CDUS, it was confirmed as acute phlebitis.

**Figure 1:**
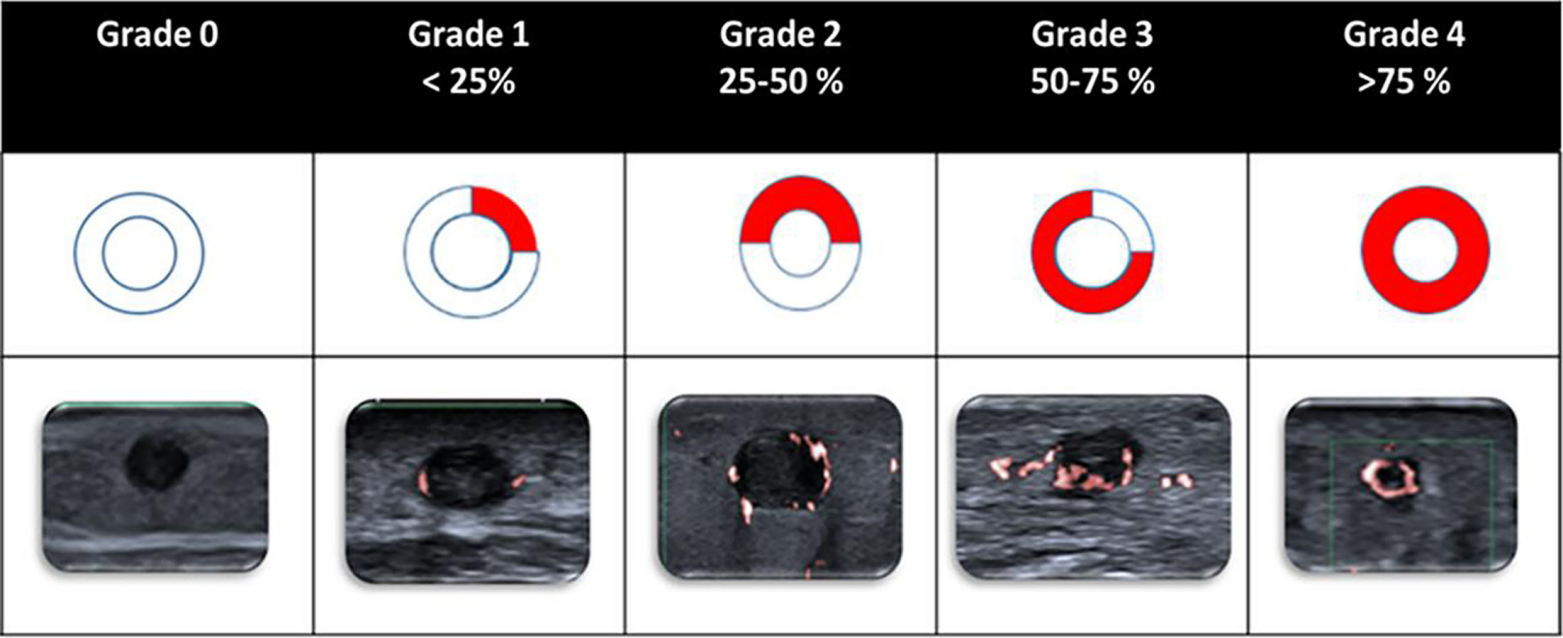
Vascular grading for venous vessel-wall signal intensity with SMI. SMI, superb microvascular imaging.

**Figure 2:**
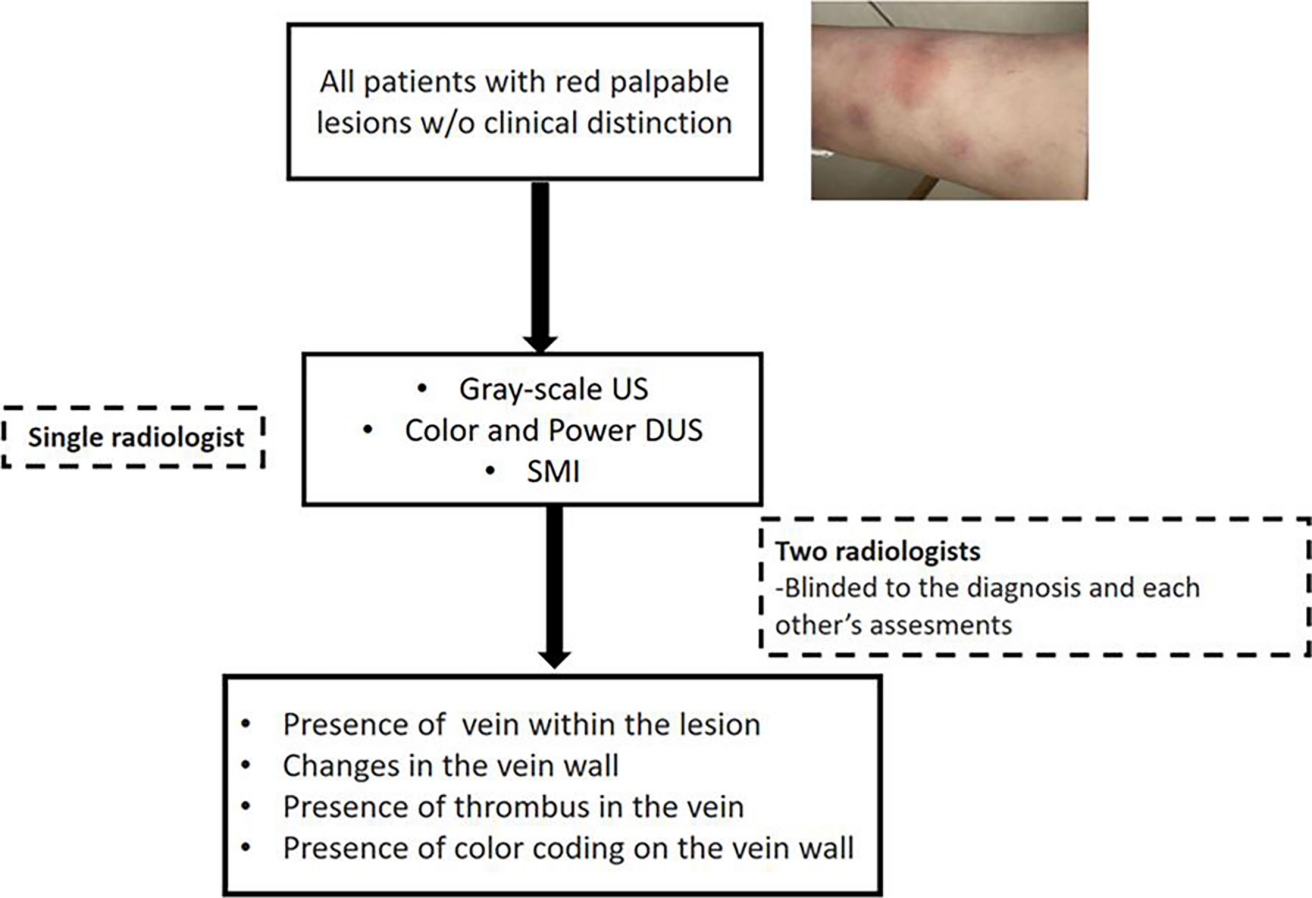
Patient evaluation flowchart. DUS, Doppler ultrasonography; SMI, superb microvascular imaging; US, ultrasonography.

The final step was to perform SMI to assess colour coding in the venous vessel wall, lumen and adipose tissue. A grading system was developed for reporting the degree of vascularisation in the venous vessel wall based on the amount of the venous vessel-wall colour coding in the axial section of the involved vessel. Venous vessel-wall signal intensity was categorised into four grades based on the proportion of the affected venous vessel-wall circumference (Grade 0: no signal, Grade 1: < 25%, Grade 2: 25%–50%, Grade 3: 50%–75% and Grade 4: >75%) (figure 1). If there was a long lesion or multiple lesions, we included the one with the highest amount of colour coding in the venous vessel wall for the analysis. The patient evaluation algorithm is summarised in figure 2.

### Interobserver and intraobserver reliability

Grey-scale US, CDUS, PDUS and SMI images were stored in a central picture archiving and communication system at the time of examination, which were later retrieved for evaluation and grading as per the above-mentioned grading system independently by two radiologists (YK and AKU), who were blinded to the diagnoses and clinical information about the subjects. 1 month after this initial grading, one of these radiologists re-evaluated the images blindly to assess intraobserver reliability.

### Statistical analysis

Data were summarised as means, SD and percentages. The proportions of categorical variables were compared using χ^2^ or Fisher exact testing based on the cell counts. Correlation between CRP levels and grading of venous vessel-wall signal intensity was analysed with Spearman’s correlation. Interobserver and intraobserver reliability were assessed by kappa statistics. The diagnostic performance of SMI in differentiating acute STM/phlebitis due to BS from non-BS aetiologies was evaluated. Sensitivity, specificity, area under the curve (AUC), positive predictive value (PPV), negative predictive value (NPV), accuracy and their 95% CIs were calculated. A p value less than 0.05 was considered significant without multiplicity adjustment. Analyses were performed using SPSS for Windows, V.20.0 (SPSS Inc., Chicago, IL, USA).

## Results

A total of 82 patients, 51 with BS and 31 with other diagnoses were evaluated. 70 patients were recruited from the rheumatology outpatient clinic, and 12 referred from the internal medicine clinic. One patient initially referred from the internal medicine clinic as a control was later diagnosed with BS. We excluded three patients with non-BS in whom red palpable lesions were identified as varicose veins. Finally, the study included a total of 43 patients with acute STM/phlebitis (26 BS/17 non-BS) and 36 patients with EN (25 BS/11 non-BS). Among the 43 patients with acute STM/phlebitis, 4 of the 26 patients with BS and 3 of the 17 patients with non-BS had acute phlebitis without thrombosis on imaging modalities.

### Patients with acute STM/phlebitis

There were 26 patients with BS (mean age: 41±12.8 years, M/F: 23/3) and 17 patients with non-BS (mean age: 43.6±12.7 years, M/F: 5/12) (table 1). 11 patients with BS (42%) had vascular involvement, 11 had (42%) eye involvement and 8 (31%) had only mucocutaneous involvement. We noticed that 15 patients with BS who were diagnosed with acute STM/phlebitis by CDUS during the study had previously been clinically diagnosed with EN. Among the 11 patients with BS with vascular involvement, 9 had lower extremity DVT, 1 had pulmonary involvement and 1 had splenic vein thrombosis. At the time of the study, 16 patients were on immunosuppressive therapy, 2 were on colchicine and 8 were off-treatment. The immunosuppressive agents included azathioprine in eight patients, mycophenolate in one patient, adalimumab in five patients (solo in one patient, combined with azathioprine in three patients, and with mycophenolate in one patient) and infliximab in two patients. Five patients were using low-dose glucocorticoids in addition to immunosuppressives.

**Table 1:** Demographic features of the patients, localisation of STM lesions and diagnoses of patients with non-BS

Variable	Patients with STM	
	BS(n=26)	Non-BS(n=17)
Mean (SD) age, years	41±12.8	43.6±12.7
Male, n (%)	23 (88.5)	5 (29)
Localisation, n (%)		
Upper extremity	4* (14.8)	4† (23.5)
Lower extremity	22 (25.2)	11 (64.7)
Chest	0	2 (11.8)
Diagnoses		
Dermatomyositis	N/A	1
Polymyositis	N/A	1
Ankylosing spondylitis	N/A	1
SLE+APS	N/A	1
Malignancy	N/A	2
Cellulitis	N/A	1
Granulomatous polyangiitis	N/A	1
Mondor’s disease	N/A	2
Venous insufficiency	N/A	3
No definite diagnosis	N/A	3

* Two patients with BS developed STM after venous cannulation.

† The diagnoses of these patients in whom acute STM developed after venous cannulation were dermatomyositis, polymyositis, cellulitis and ankylosing spondylitis.

APS, antiphospholipid syndrome; BS, Behçet syndrome; N/A, not applicable; SLE, systemic lupus erythematosus; STM, superficial thrombophlebitis.

The most common localisation of acute STM/phlebitis was lower extremity in both groups (22/26 for BS and 11/17 for non-BS). STM was on the chest wall in two patients with non-BS and on the upper extremity in four patients in each group. Acute STM/phlebitis in the upper extremity developed after venous cannulation in two of the four patients with BS and all of the four patients with non-BS. The demographics of the patients, localisation of STM lesions and diagnoses of non-BS patients are summarised in table 1.

### Imaging results of patients with acute STM/phlebitis

**Figure 3:**
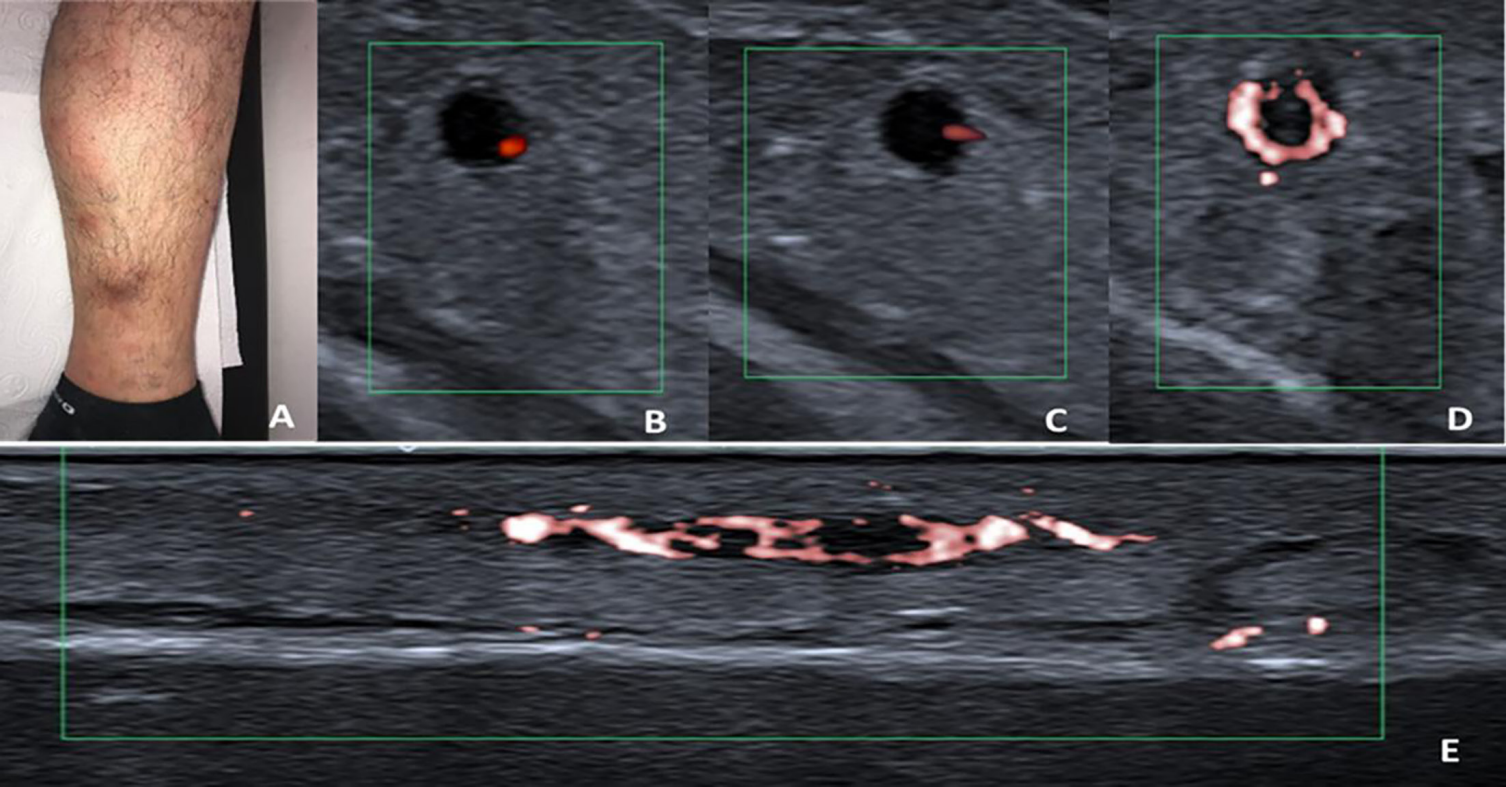
An adult patient with BS presented with acute red, painful nodule-like lesion (**A**) in the posterior part of the left cruris was evaluated. (**B**) Colour and (**C**) power Doppler images show a filling defect compatible with thrombus in the vein, and spot colour coding is seen on the venous vessel wall. (**D**) On the other hand, SMI image shows grade 4 signal intensity in the axial plane of the venous vessel wall, which suggests vasculitic involvement. (**E**) In the longitudinal plane, segmental involvement of the venous vessel wall is observed. BS, Behçet syndrome; SMI, superb microvascular imaging.

Among the 43 patients with acute STM/phlebitis, SMI detected colour-coded flow signal in the venous vessel wall in 24 patients (21 patients with BS and 3 patients with non-BS). The frequency of the presence of colour-coded flow signal on the venous vessel wall was significantly higher in patients with BS than in patients with non-BS (21/26 (81%) vs 3/17 (18%), p<0.0001). According to the grading system, at least grade 2 or higher venous vessel-wall signal was detected in 16/21 patients with BS (76%) and 4 of them had full-circle venous vessel-wall signal with a halo shape on the venous vessel wall (figure 3D). Among the four patients with BS with phlebitis, one had grade 4 and the other had grade 1 venous vessel-wall signal, whereas the other two did not show any colour-coded signal on the venous vessel wall with SMI.

Among the three patients with non-BS with colour-coded signal intensity on the venous vessel wall, one had grade 3 signal intensity, while the other two had grade 1 signal intensity. None of these patients had a history of chronic disease. The causes of acute STM were venous insufficiency, venous cannulation and cellulitis in one patient each. Venous cannulation had been performed for intravenous antibiotics due to pneumonia. Grade 3 venous vessel signal intensity was observed in the patient with cellulitis, while the other two patients with acute STM lesions had grade 1 venous vessel signal intensity (table 2).

**Table 2:** Presence and grade of venous vessel-wall signal intensity according to SMI in BS and control patients

Venous vessel-wall signal intensity grading with SMI, n (%)	Patients with BS(n=26)	Patients with non-BS(n=17)
No signal*	5 (18.5)	14 (82.4)
Grade 1	6 (22.2)	2 (11.8)
Grade 2	9 (35)	0
Grade 3	2 (7.4)	1
Grade 4	4 (14.8)	0

* Having no vessel-wall signal was significantly more common among patients with non-BS compared with patients with BS, p=0.0001.

BS, Behçet syndrome; SMI, superb microvascular imaging.

The CRP level was above the cut-off in 15 of the 26 patients with BS, with a mean (SD) CRP level of 20±18 mg/L. The presence of venous vessel-wall signal intensity was somewhat more frequent in patients with an elevated CRP than in those without (14/15 vs 7/11, p=0.057). Among patients with venous wall signal intensity and negative CRP, the grade of signal intensity was grade 1 in one patient, grade 2 in five patients and grade 4 in one patient. Among patients with venous wall signal intensity and positive CRP, the grade of signal intensity was grade 1 in five patients, grade 2 in four patients, grade 3 in two patients and grade 4 in three patients. There was no correlation between grading of venous vessel-wall signal intensity in patients with BS and CRP levels (r=0.24, p=0.22).

The interobserver reliability (κ=0.87, p<0.001) and intraobserver reliability (κ=0.81, p<0.001) were good for grading the signal intensity on the venous vessel wall using SMI.

Of note, among the 43 patients with STM/phlebitis, CDUS and PDUS detected spot-like colour coding on the venous vessel wall in only 2 patients with BS with STM (figure 3B,C).

### Specificity and sensitivity of SMI for patients with BS versus patients with non-BS with STM

We looked at whether SMI may help in the differential diagnosis of patients presenting with acute STM/phlebitis. According to the SMI grading, the presence of venous vessel-wall signal intensity had a sensitivity of 80.7% and a specificity of 82.3%, with an AUC of 0.816 (95% CI 0.678 to 0.954) in differentiating acute STM/phlebitis due to BS from non-BS aetiologies. The PPV was 87.5%, the NPV was 73.7% and the accuracy was 81.4%.

### Imaging results of patients with EN

There were 25 patients with BS and 11 patients with non-BS with EN. CDUS, PDUS or SMI did not detect any colour-coded signal in the lesions.

## Discussion

In this study, we evaluated colour-coded signals on the venous vessel wall in patients with BS with acute STM/phlebitis to detect vessel-wall vascularisation, which seems to indicate inflammation within the vessel wall, and compared these to controls with acute STM/phlebitis due to other causes. We found that 81% of patients with BS with STM/phlebitis (21 out of 26) had venous vessel-wall signals, and 76% of these patients (16 out of 21) exhibited grade 2 or higher signal intensity on the venous vessel wall with SMI. In contrast, no colour coding was detected in 82% of patients with non-BS with STM/phlebitis (14 out of 17), showing a sensitivity of 80.7% and a specificity of 82.3% in differentiating BS-related STM/phlebitis from other causes of STM/phlebitis. These results suggest that SMI may have potential for use in differentiating inflammatory venous lesions from non-inflammatory ones and deserve studying further in this respect.

A number of studies showed increased vein wall thickness by magnetic resonance^26 27^ and US in patients with BS, even in the absence of vascular involvement.^28^,^34^ "Although increased vein wall thickness in BS is consistently reported, its actual significance — and whether it reflects active inflammation or the sequelae of prior vessel-wall inflammation — remains uncertain. Identifying an imaging method showing inflammation of the vein wall, similar to positron emission tomography (PET)/CT scans for the arterial wall, would be very useful in clinical practice for determining active disease and guiding treatment decisions.

SMI is increasingly being investigated for assessing vascularisation in several conditions.^35^ On the other hand, there is little data on its use as an indicator of vessel-wall inflammation. A recent study reported encouraging findings with SMI in assessing disease activity in Takayasu arteritis.^25^ The authors examined carotid arteries in 45 active and 51 inactive Takayasu patients according to the National Institutes of Health score. SMI was superior to CDUS in visualising vessel wall, vessel lumen and vessel borders, and in detecting neovascularisation within the vessel wall. SMI grades were higher in active patients than in inactive patients and Takayasu patients with vascularisation detected by SMI were 4.7 times more likely to have active disease. Moreover, in a patient with Takayasu arteritis, SMI-detected vessel-wall inflammation in the carotid artery was colocalised with 18-fluorodeoxyglucose uptake in PET/CT, both of which disappeared after 6 months of glucocorticoid therapy.^36^ Additionally, we previously reported bilateral SMI-detected signal in the temporal artery walls in a patient with giant cell arteritis, which disappeared in one side and weakened in the other artery after 1 month of immunosuppressive therapy.^37^

Vessel-wall inflammation is considered to play a key role in the pathogenesis of BS. Direct evidence for this is the presence of inflammatory cell infiltration within the vessel wall in nodular lesions that were diagnosed as STM.^12 16^ On the other hand, histopathological studies investigating pulmonary artery aneurysms, central nervous system involvement and gastrointestinal involvement typically detected perivascular inflammation rather than inflammation within the vessel wall.^13^,^15^ These differences may be due to the timing of the biopsies, the stage of inflammation, prior treatment before the biopsy and the challenges pathologists face in interpreting biopsies as well as the possibility of distinct pathologies in different organ involvements.

The frequency and intensity of SMI-detected colour-coded signals were more pronounced in acute STM/phlebitis lesions of patients with BS compared with controls. However, 5 of the 26 patients with BS did not show any signals, while 3 of the 17 controls showed signals. The timing of imaging might explain the failure to detect inflammation in these five patients with BS, as older or very early lesions may exhibit less inflammation. Additionally, 42% of patients with BS had a negative CRP, which is a less expected finding for a patient with STM, and a greater number of patients with grade 0 signal intensity were among those with a negative CRP, further supporting this observation. Moreover, the finding that two patients with BS with acute phlebitis, which may represent an early or less inflamed form of STM, did not show any signal also supports this conclusion. Even though three patients with non-BS had colour-coded signals, only one of them had grade 3 signals. The weak colour-coded signals in two patients with non-BS might be explained by neovascularisation due to ischaemia of the venous vessel wall and/or a secondary local inflammatory response from venous thrombosis. Indeed, a study exploring histopathology of varicose veins in 20 patients showed damaged endothelial areas, a high density of the vasa vasorum in the tunica media and tunica adventitia and neoangiogenesis compared with normal veins. In two samples, extravasation of inflammatory cells was also observed in the vasa vasorum, with infiltration into the vein wall in one.^38^ The grade 3 signal observed in the patient with cellulitis seems to be an expected finding, as the infection of soft tissues adjacent to the vein has likely led to acute phlebitis.

We did not observe SMI signals in any of the EN lesions in either group. This is an intriguing finding because previous studies on the histopathology of EN lesions had suggested phlebitis, venulitis and even thrombosis within some of these lesions in addition to a septal or mixed type panniculitis.^12^ This raises the question of whether EN and STM lesions are not always distinct lesions in BS and whether there may be lesions with overlapping features. This finding deserves further research exploring the correlation of imaging findings, including SMI and histopathology of these lesions.

Our study has some limitations. First, we recruited a relatively small and inhomogeneous control group. However, it was challenging to include a control group with a uniform disease like BS, where STM is frequently observed during the disease course. In future studies, the inclusion of control groups consisting of patients with conditions known to be associated with STM—such as malignancies, inflammatory bowel disease or other vasculitides—would be valuable to further validate the specificity and utility of SMI in detecting vessel-wall inflammation in BS. Second, a single radiologist performed all imaging; however, the recorded images were later evaluated blindly by two radiologists. Finally, we did not consider the time between lesion onset and the evaluation of the lesions, which may lead to variability in grading signal intensity.

In this study, we evaluated EN and STM lesions in detail using SMI to gain a deeper understanding of these lesions and to explore the potential of SMI for use in research and clinical practice. Assessment of disease activity in vascular involvement of BS remains challenging with the current clinical, laboratory and imaging biomarkers.^39^ The increased signals detected by SMI on vessel walls of STM lesions seem to support the presence of vessel-wall inflammation. Further work is needed to support the use of SMI in patients with BS, including investigation of the correlation among SMI findings, histopathology and disease activity. Moreover, the increased signals on the vessel wall of STM observed in this study should be confirmed in different vascular lesions, especially in larger veins in BS, with appropriate controls. If these studies support the reliability of SMI in showing vessel-wall inflammation in BS, it may become an important tool that will guide treatment decisions in patients with vascular involvement. Additionally, its role in the differential diagnosis of patients with recurrent thrombosis needs to be investigated. This is an important problem since several patients with BS present with vascular involvement before other manifestations that would lead to a diagnosis of BS.^1 3 5 40^ Lack of immunosuppressive treatment in such patients results in recurrences that may lead to post-thrombotic syndrome. Even if a certain diagnosis of BS is not made, identifying inflammation of the vein wall and initiation of immunosuppressive treatment may provide better long-term outcomes than giving anticoagulants alone. In conclusion, the evaluation of the vessel wall with SMI offers a promising approach for patients with BS as it is an inexpensive, non-invasive and reliable imaging method that does not require a contrast agent.

## Data Availability

All data relevant to the study are included in the article or uploaded as supplementary information.
